# Is timing everything? Age-related differences in the use of temporal agency cues

**DOI:** 10.1007/s00426-026-02395-2

**Published:** 2026-10-08

**Authors:** Victoria K. E. Bart, Martina Rieger

**Affiliations:** Institute of Psychology, UMIT TIROL - Private University for Health Sciences, Hall in Tirol, Austria

## Abstract

Sense of agency (SoA) is the subjective experience of controlling one’s own actions and their effects. To infer SoA people rely, among other cues, on temporal cues such as temporal contiguity (i.e. the closeness in time between action and effect) and temporal expectation (i.e. the match between expected and actual timing of an effect). We investigated whether the use of temporal cues and their interplay differ between younger (18–28 years) and older (65–75 years) adults. In the experiment, participants first completed adaptation blocks to form expectations regarding the timing of an effect. In adaptation blocks, actions were followed either by immediate (immediate effect condition) or by delayed (delayed effect condition) effects. In subsequent test blocks, the action-effect delay was varied, and participants SoA over the effect was assessed. Across both age groups, SoA increased, the more the timing of the actual effect corresponded to the expected effect, indicating that temporal expectation is used as primary agency cue. However, in younger (but not in older) adults, the change in SoA as a function of delay was steeper in the immediate effect condition than in the delayed effect condition. This may indicate that temporal contiguity and temporal expectation have reinforced each other in the immediate effect condition but counteracted each other in the delayed effect condition. Thus, whereas both age groups primarily rely on temporal expectation, younger adults may additionally integrate temporal contiguity when inferring SoA.

**Supplementary Information:**

The online version contains supplementary material available at https://doi.org/10.1007/s00426-026-02395-2.

## Public significance statement

Understanding how people experience agency over their actions and effects is crucial for everyday functioning and well-being. This study shows that younger and older adults judge agency based on whether an action’s effect occurs at the expected time. Only younger adults additionally rely on the closeness in time between action and effect. These findings reveal age-related differences in the experience of agency, which may inform the design of technologies to better support the experience of agency across the lifespan.

**Supplementary Information:**

The online version contains supplementary material available at https://doi.org/10.1007/s00426-026-02395-2.

## Introduction

Sense of agency (SoA) is the subjective experience of having control over one’s own actions and their effects in the outside world (Haggard & Tsakiris, 2009). People rely on various cues to judge their SoA (see Moore & Fletcher, 2012 for a review), including temporal cues, i.e. the temporal relationship between actions and their resulting effects (Vuorre, 2017). Two temporal cues may be distinguished. Temporal contiguity refers to the observation that SoA is stronger when action and effect are closer together in time than when they are not (Farrer, et al., 2013; Ruess et al., 2017). Temporal expectation refers to the observation that SoA increases when the expected timing and the actual timing of an effect match (Haering & Kiesel, 2015, 2016). The reliance on those cues may vary depending on age (Mariano et al., 2024; Nobusako et al., 2024). However, so far only the use of temporal contiguity, but not the use of temporal expectation has been investigated with respect to age-related differences. Thus, the aim of the present study was to investigate whether the use of temporal cues (temporal contiguity and temporal expectation) and their interplay differ between younger (18–28 years) and older (65–75 years) adults.

In cue integration models it is proposed that people rely on different sources of information (agency cues) to judge their SoA (Moore & Fletcher, 2012; Synofzik et al., 2008, 2009). These cues may include, among others, thoughts or beliefs prior to the action (Pronin et al., 2006; Wegner & Wheatley, 1999) and contextual cues (e.g., the presence of someone else, who could have caused the effect, Lafleur et al., 2020). Further, affective cues (e.g. the affective valence of the effect or the affective state of the agent, Gentsch & Synofzik, 2014) and sensorimotor cues (e.g. a match between actual and internally predicted effect, Krugwasser et al., 2019) are used to infer SoA. Rather than relying on a single source of information, people integrate these available cues to infer whether an observed effect was caused by their own action. The weight assigned to each cue depends on its reliability and relevance in the given situation, and this evaluation is reflected in the strength of one’s SoA (Moore & Fletcher, 2012; Synofzik et al., 2009).

Temporal cues, i.e. the temporal relationship between an action and its effect (Vuorre, 2017; Wen, 2019), play also an important role in this evaluation process. One such temporal cue is the temporal contiguity between action and effect (Farrer et al., 2013; Ruess et al., 2017). When action and effect are close together in time, they are perceived as causally related (Reed, 1992). Presumably, the same mechanisms underlying perceived causality contribute to SoA (De Vignemont & Fourneret, 2004). Accordingly, it has been observed that SoA is higher when action and effect are close together in time than when a delay is present between action and effect (Dewey & Carr, 2013; Ebert & Wegner, 2010; Farrer et al., 2013; Kawabe, 2013; Ruess et al., 2017; Sato & Yasuda, 2005; Wen et al., 2015).

However, in situations in which delayed effects occur people can still experience strong SoA if the delay matches their prior expectation (Bart et al., 2024; Haering & Kiesel, 2015, 2016). It has been proposed that repeatedly performing an action leads to the formation of a bidirectional association between the action and its effect (ideomotor theory, Hommel, 2003; Hommel et al., 2001). This association may also include the delay between action and effect (Dignath et al., 2014; Dignath & Janczyk, 2017). When the action is performed again, this delay becomes automatically retrieved, i.e. expectations (based on internal predictions) about the timing of the effect in relation to the action are formed (Dignath et al., 2014; Dignath & Janczyk, 2017). SoA is higher when the timing of the effect matches the expectation compared to when the effect appears earlier or later, suggesting that temporal expectation is used as agency cue (Bart et al., 2024; Haering & Kiesel, 2015, 2016).

So far, the contribution of the different temporal agency cues (temporal contiguity and temporal expectation) is relatively well understood in younger adults (from 19 to 39 years depending on the study). Particularly, it seems that younger adults use temporal contiguity as an agency cue (e.g. Dewey & Carr, 2013; Ebert & Wegner, 2010; Farrer et al., 2013), when it is the only available temporal cue or when they have no prior temporal expectations regarding the delay between action and effect. However, when prior expectations regarding the timing of a certain effect in relation to the action that produces it exist, temporal expectation has a stronger impact than temporal contiguity (Bart et al., 2024; Haering & Kiesel, 2015, 2016).

Studies suggest that aging affects the reliability of sensory and motor signals differently. Specifically, age-related structural and functional changes in frontostriatal networks have been proposed to reduce the precision of external sensory signals, increasing reliance on comparatively less noisy internal sensorimotor predictions (Wolpe et al., 2016; Mariano et al., 2024). This also applies to the processing of temporal information. Aging seems to be associated with increased noise and temporal uncertainty (Turgeon et al., 2016). Such increased variability in temporal representations seems to be compensated by an increased reliance on temporal prediction (Otsuka et al., 2025). From a cue integration perspective, this may imply that older adults might place relatively more weight on internal predictive cues, such as temporal expectation, and relatively less weight on external sensory cues such as temporal contiguity.

However, the contribution of temporal expectation and temporal contiguity to SoA has hardly been investigated in older adults. So far it is known that the general level of SoA is reduced in older (between 54 and 72 years) compared to younger (between 17 and 34 years) adults (Cioffi et al., 2017). Further, whereas, the use of temporal expectation in older adults has not yet been investigated, studies indicate that younger and older adults may differ in the use of temporal contiguity as an agency cue (Bart & Rieger, 2026; Mariano et al., 2024; Nobusako et al., 2024). However, those few studies have shown mixed results. In some studies, reduced SoA has been observed with increasing action-effect delay in younger adults (~ 18–28 years), whereas SoA remained relatively unaffected in older adults (~ 54–79 years) (Bart & Rieger, 2026; Mariano et al., 2024; Metcalfe et al., 2010). This may indicate that older adults are less likely to use temporal contiguity as an agency cue compared to younger adults. In contrast, Nobusako et al. (2024) reported a stronger reduction in SoA with increasing action-effect delay in older (65–83 years) compared to younger adults (20–25 years).

Those diverging results may be explained by the cue integration theory. In the study by Nobusako et al. (2024) temporal contiguity was the only available agency cue, whereas in other studies additional (internal) agency cues (i.e. cues based on predictions generated by internal forward models, for instance internal sensorimotor cues like a match between actual and predicted effect, cf. Bart & Rieger, 2026) were available. Thus, in the absence of internal agency cues older adults may rely more on external sensory cues such as temporal contiguity, maybe even in a more rigid way than younger adults. However, as soon as internal cues become available, older adults may perceive them as more reliable, resulting in a lower confidence in temporal contiguity as agency cue (Bart & Rieger, 2026; Mariano et al., 2024).

To investigate age-related differences in the use of temporal agency cues, we adapted an experimental paradigm originally developed by Haering and Kiesel (2015), which allows to dissociate between the contribution of temporal contiguity and temporal expectation to SoA. Younger and older adults first completed adaptation blocks in which their action was consistently followed either by immediate effects (0 ms after the action, immediate effect condition) or by delayed effects (600 ms after the action, delayed effect condition). Accordingly, depending on the condition, different expectations about the timing of an effect in relation to the action that produces it were generated. In subsequent test blocks, the delay between action and effect was systematically varied (0 ms, 150 ms, 300 ms, 450 ms, 600 ms). Participants were told that during test blocks their action usually causes the effect, but that in some trials the effect is automatically generated by the computer independent of their action. Participants were asked to rate how much control they felt over the effect.

For younger adults, we expected to replicate the result that temporal expectation has a stronger impact than temporal contiguity (Bart et al., 2024; Haering & Kiesel, 2015, 2016). Accordingly, control ratings should be higher, the less the actual timing of the effect deviates from the expected timing of the effect. Thus, in the immediate effect condition control ratings should be highest when effects occur immediately, whereas in the delayed effect condition control ratings should be highest when the delay of the effect corresponds to the delay used in the adaptation blocks. However, we expected that temporal contiguity still plays a role as this has been observed previously (Bart et al., 2024). In the immediate effect condition, participants expect the effect at 0 ms. Thus, both temporal expectation and temporal contiguity are fulfilled at short delays. Accordingly, those two cues should reinforce each other, resulting in a steeper decline in SoA ratings across increasing action-effect delays (0, 150, 300, 450, 600 ms). In contrast, in the delayed effect condition, in which participants expect the effect at 600 ms, temporal expectation is met at longer delays, but temporal contiguity is violated. The opposing influence of these cues is expected to result in a flatter SoA curve across delays compared to the immediate effect condition (see Bart et al., 2024 for such results).

For older adults, we expected that, just like younger adults, temporal expectation is used as agency cue. Accordingly, in older adults (just like in younger adults), control ratings should be higher, the less the actual timing of the effect deviates from the expected timing of the effect. However, in contrast to younger adults, temporal contiguity may not be used/may be used less in older adults (cf. Bart & Rieger, 2026, Mariano et al., 2024) and may therefore have less influence on SoA in older adults. Accordingly, the difference in the steepness of the SoA curves between the immediate and the delayed effect condition is expected to be smaller in older adults than in younger adults.

## Method

### Participants

The study employed a convenience sample. Undergraduate psychology students conducted the data collection as part of their course work. Each student recruited both younger and older adults from their own personal network. Originally 160 participants took part in the study (80 younger adults and 80 older adults). Participants were included in the study if they met the following criteria: (a) an age between 18 and 28 years for the younger adults or between 65 and 75 years for the older adults (b) no self-reported history or current diagnosis of motor impairments or neurological/psychiatric diseases, and (c) no use of psychoactive medication. All participants had normal or corrected-to-normal vision. Younger and older adults were matched based on education. They were randomly assigned to one of two conditions, which differed in the temporal expectation that was induced regarding the appearance of the effect (immediate effect condition or delayed effect condition, cf. Haering & Kiesel, 2015 for such an approach). The study received ethical approval from the Research Committee for Scientific Ethical Questions (RCSEQ) at UMIT TIROL. All participants gave written informed consent.

27 participants (10 younger adults, 17 older adults) had to be excluded from analyses for reasons stated below. The final sample consisted of 70 younger adults (immediate effect condition: *N* = 37, delayed effect condition: *N* = 33) and 63 older adults (immediate effect condition: *N* = 32, delayed effect condition: *N* = 31). Demographic data as well as data of the background neuropsychological screening (see supplemental material Sect. 2 for a detailed description of the neuropsychological tests) of older and younger adults depending on temporal expectation (immediate effect condition, delayed effect condition) are shown in Tables 1 and 2, respectively. Whereas demographic data hardly differed depending on age and temporal expectation, as can be expected younger adults performed better than older adults on most neuropsychological tests (see supplemental material Sects. 1 and 3 for the statistical comparisons).

We used MorePower 6.0.4 (Campbell & Thompson, 2012) to calculate the required sample size for several effects of interest (e.g., all main effects and all interactions including the factor age) in a mixed ANOVA model with one within-participants factor and two between-participants factors. Statistical significance was set at *p* <.05 and the power was set at 0.9. We choose a medium effect size (η_p_² = 0.1) based on previous studies (cf. Bart et al., 2024, 2026; Mariano et al., 2024). The required sample size ranged between 40 and 104 (i.e. between 10 and 26 for each between-participants condition), depending on the effect of interest.

**Table 1:** Descriptive statistics of demographic data depending on age (younger adults, older adults) and temporal expectation (immediate effect condition, delayed effect condition)

	younger adults		older adults	
	immediate effect condition	delayed effect condition	immediate effect condition	delayed effect condition
Sex (women/men)	23/14	20/13	19/13	18/13
Handedness (right/left/both)	33/1/3	29/3/1	28/3/1	30/1/0
Age *M* (*SD*)	23.5 (2.9)	23.3 (3)	67.6 (2.7)	69.8 (3.6)
Years of formal education *M* (*SD*)	14.5 (2.2)	14.4 (2.1)	14.2 (3.1)	13.5 (3.9)
Hours sport/week *M* (*SD*)	7.8 (7.8)	6.3 (6.3)	10.8 (9.9)	8.7 (8.8)
Hours cultural activity/week *M* (*SD*)	1.2 (2.2)	1.1 (1.7)	2.1 (3.6)	1.1 (2)
Hours intellectual activity/week *M* (*SD*)	5.7 (8.3)	4.4 (4.2)	11.9 (8)	9.8 (7.1)

**Table 2:** Descriptive statistics (means and standard deviation in parenthesis) of the data of the background neuropsychological screening, depending on age (younger adults, older adults) and temporal expectation (immediate effect condition, delayed effect condition)

	younger adults		older adults	
	immediate effect condition	delayed effect condition	immediate effect condition	delayed effect condition
Visual working memory	4.1 (2)	4.5 (1.6)	2.4 (1.7)	2.1 (1.4)
Verbal working memory	7.8 (1.9)	8 (1.9)	6.5 (2.1)	6.4 (1.5)
Verbal fluency	16.2 (5.2)	16.8 (4.8)	15.3 (5.3)	14.5 (6.3)
Attention: number of processed target stimuli	88.7 (17.5)	90.7 (16.8)	57.8 (16.9)	52.5 (16.4)
Attention: concentration performance	81.1 (16.8)	84.5 (15.7)	49 (16)	45.8 (15.2)
Attention: error percent	8.5 (6.8)	6.6 (6.4)	15 (14.5)	12.9 (9.2)
Spatial ability	27.7 (5.6)	28.7 (4.9)	20.2 (7.1)	20.4 (6.7)

### Procedure

Participants were tested individually. Each session began with the collection of demographic information and the administration of the brief background neuropsychological screening including tests evaluating selective attention, verbal fluency, verbal and visual working memory, and spatial ability. For a detailed description of those tests see supplemental material, Sect. 2.

Afterwards, participants completed the SoA task. Participants were seated in front of a Toshiba Tecra R950 laptop (screen: 15.6″, vertical refresh rate: 100 Hz, resolution: 1600 × 900 pixels). The experiment was programmed with the open-source experimental software OpenSesame 3.3.12 (https://osdoc.cogsci.nl; Mathôt et al., 2012) and was based on the experimental procedure of Haering and Kiesel (2015).

**Fig. 1:**
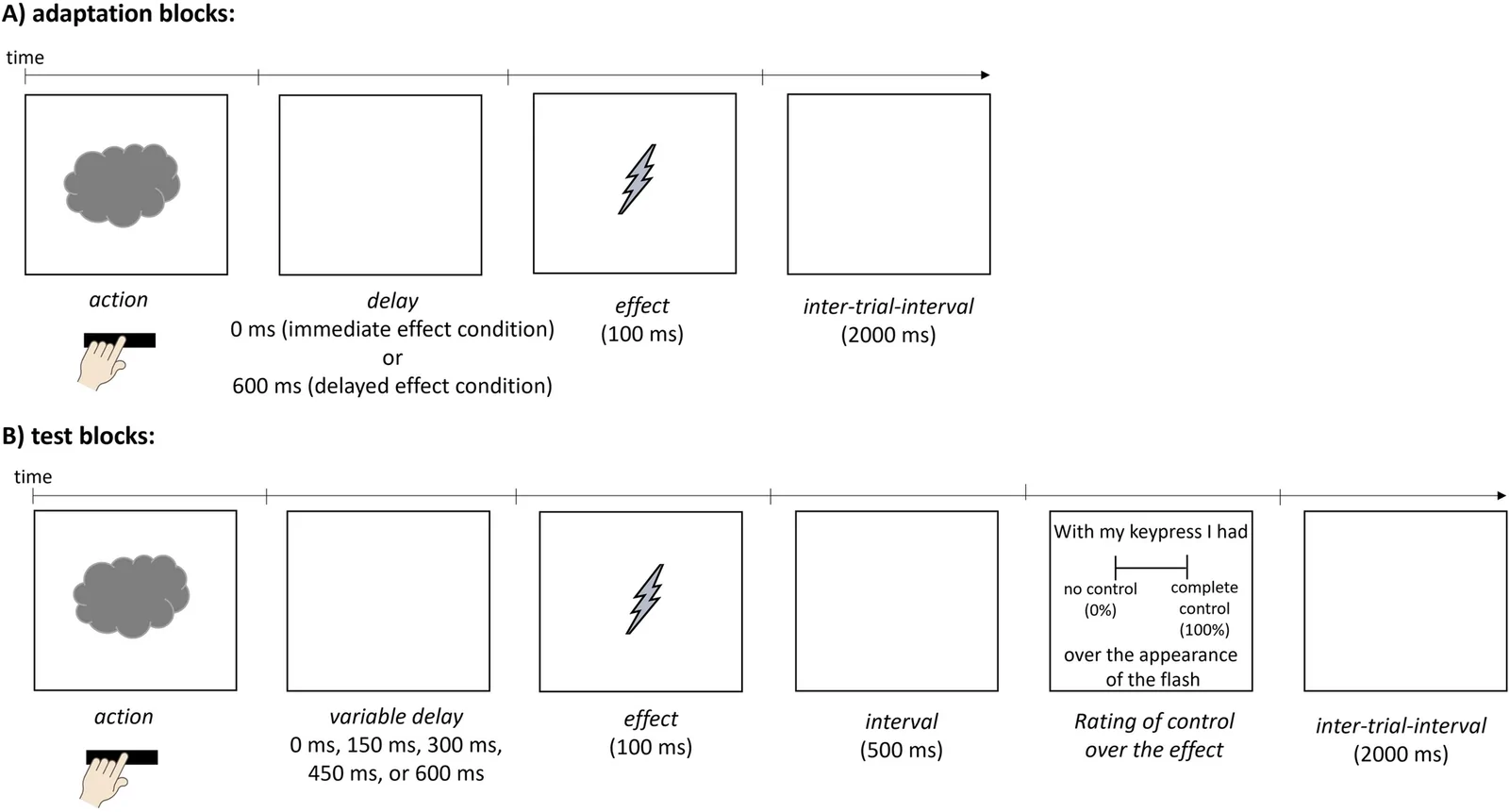
Depiction of the trial procedure in adaptation blocks (50 trials per block, panel A) and test blocks (50 trials per block, panel B). Note that in test blocks the flash occurred in 30 trials with its usual delay (i.e., the delay used during the adaptation blocks: either 0 ms or 600 ms depending on the condition) and with any of the other delays in 5 trials per test block

The experiment consisted of adaptation and test blocks. The trial procedure in each block type can be seen in Fig. 1. In adaptation blocks, each trial began with the presentation of a grey cloud (size: 7.5 cm x 5.6 cm) centered on a black background. Participants were instructed to press the spacebar on the keyboard with the index finger of their left hand as soon as the cloud appeared. This response was followed either immediately (delay of 0 ms, immediate effect condition) or after a delay of 600 ms (delayed effect condition) by the presentation of a yellow flash in the center of the screen (4 cm x 8.7 cm; presentation time: 100 ms). After an inter-trial-interval of 2000 ms the next trial started. Prior to each adaptation block, participants were informed that their response would cause the flash. Each adaptation block contained 50 trials.

Test blocks followed the same general trial procedure, except for the following differences: Instead of using a constant action-effect delay, the flash appeared after a variable delay (0 ms, 150 ms, 300 ms, 450 ms, or 600 ms). At the beginning of each test block participants were told that during the following block their response usually causes the flash, but that in some trials the flash is automatically generated by the computer independent of their response. This was done to create ambiguity regarding the source of the effect. In each trial, 500 ms after the flash disappeared, participants were asked to rate how much control they experienced over the flash via mouse click on a visual analog scale (11.5 cm) from “no control” (0%) to “complete control” (100%). This resulted in the appearance of a red cross (0.4 × 0.4 cm, presentation time: 500 ms) at the point of the scale where participants clicked at. After an inter-trial-interval of 2000ms the next trial started. Each test block contained 50 trials. The flash occurred in 30 trials with its usual delay (i.e., the delay used during the adaptation blocks: either 0 ms or 600 ms depending on the condition). The remaining 20 trials contained the four alternative delays, each presented five times.

**Fig. 2:**
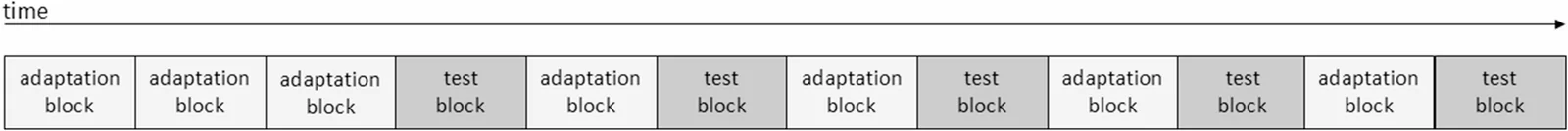
Depiction of the overall block sequence

For an overview of the block sequence see Fig. 2. Participants first completed three initial adaptation blocks (A). Afterwards they performed five test blocks (T), each separated by an adaptation block. This resulted in an A-A-A-T-A-T-A-T-A-T-A-T pattern. The study took, including breaks, approximately 1–2 h.

### Data analysis

We excluded twenty-three participants (10 younger adults, 13 older adults) from analyses because the standard deviation of their control ratings over all trials in the test blocks was less than 1. This indicated that they did not answer thoroughly but clicked at the same point of the visual analogue scale in every trial. Further, four participants (all older adults) were excluded because they failed to adhere to the instructions in at least one of the tests of the neuropsychological background screening.

We calculated mean control ratings per participant separately for each delay. As described above, the flash appeared with its usual delay (either 0 ms or 600 ms depending on the group) in 30 trials and with any other delay in 5 trials per test block. To ensure that the mean control ratings for the different delay conditions were based on an equal number of trials, we randomly selected five out of the 30 trials with the usual delay in each test block to compute the mean control ratings. This resulted in a total of 25 trials per delay (five test blocks with five trials per delay each).

The data were analyzed using ANOVAs (see results section). If Mauchly’s test indicated that the assumption of sphericity was violated, Greenhouse-Geisser corrected *F*-values, *p*-values, and Greenhouse-Geisser’𝜀 are reported. Significance values for post hoc comparisons were adjusted for multiple testing using Sidak correction. When several post hoc comparisons are reported together, minimum (*p*_min_) or maximum p-values (*p*_max_) are reported. The data can be retrieved from the open science framework, https://osf.io/yghmb/.

## Results

### Differences in control ratings depending on age, temporal expectation, and delay

**Fig. 3:**
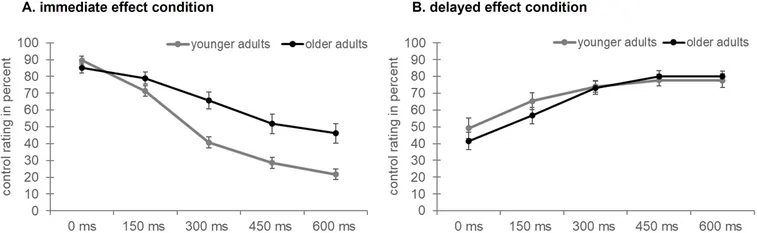
Means and standard errors of control ratings as a function of age (younger adults, older adults) and delay (0 ms, 150 ms, 300 ms, 450 ms, 600 ms), separately for the temporal expectation conditions (immediate effect condition in panel A and delayed effect condition in panel B)

An ANOVA with the between-participants factors age (younger adults, older adults) and temporal expectation (immediate effect condition, delayed effect condition) and the within-participants factor delay (0 ms, 150 ms, 300 ms, 450 ms, 600 ms) was performed on the control ratings. Means and standard errors of control ratings as a function of age (younger adults, older adults) and delay (0 ms, 150 ms, 300 ms, 450 ms, 600 ms), separately for the temporal expectation conditions (immediate effect condition, delayed effect condition) can be seen in Fig. 3. The significant main effect of delay, *F*(1.45, 187.28) = 10.8, *p* <.001, η^2^_p_ = 0.08, ε = 0.36 and the significant main effect of temporal expectation, *F*(1, 129) = 9.13, *p* =.003, η^2^_p_ = 0.07, were modified by a significant interaction between temporal expectation and delay, *F*(1.45, 187.28) = 159, *p* <.001, η^2^_p_ = 0.55, ε = 0.36. In the immediate effect condition, control ratings were highest at the 0 ms delay and significantly decreased with increasing delay (all *p* <.001). In contrast, in the delayed effect condition control ratings were higher at the 600 ms, 450 ms, 300 ms, and 150 ms delay than at the 0 ms delay (all *p* <.001), higher at the 600 ms, 450 ms, and 300 ms than at the 150 ms delay (all *p* <.001), and higher at the 450 ms than at the 300 ms delay (*p* =.005). All other comparisons were not significant (*p*_min_ = 0.11).

The significant main effect of age, *F*(1, 129) = 3.95, *p* =.049, η^2^_p_ = 0.03, was modified by a significant interaction between age and temporal expectation, *F*(1, 129) = 7.61, *p* =.007, η^2^_p_ = 0.06 and by a significant interaction between age and delay, *F*(1.45, 187.28) = 9.55, *p* =.001, η^2^_p_ = 0.07, ε = 0.36. Taken together, those interactions indicate that control ratings were lower in younger than in older adults at the 300 ms, 450 ms, and 600 ms delay in the immediate effect condition (*p*_max_ < 0.001), but did not significantly differ at the 0 ms and 150 ms delay in the immediate effect condition (*p*_min_ = 0.18) nor at any delays in the delayed effect condition (*p*_min_ = 0.15). However, the three-way interaction between age, temporal expectation, and delay was not significant, *F*(1.45, 187.28) = 2.2, *p* =.13, η^2^_p_ = 0.02, ε = 0.36.

### Correlation coefficients and regression slopes

We were interested in the correlation coefficients between delay and control rating. Pearson correlation coefficients were computed separately for each participant In Table 3 means of correlation coefficients (Fishers’z transformation and back-transformation were used to average correlation coefficients) between delay and control rating, depending on age (younger adults, older adults) and temporal expectation (immediate effect condition, delayed effect condition) are depicted. In both, younger and older adults, a negative correlation coefficient was observed in the immediate effect condition, whereas a positive correlation coefficient was observed in the delayed effect condition. To check whether the size of the correlation coefficients (correlation coefficients were Fisher’s z transformed for this purpose) differed depending on age and temporal expectation, we inverted the correlation coefficients in the delayed effect condition by multiplying them with − 1 (cf. Haering & Kiesel, 2015) and computed an ANOVA with the between-participants factors age (younger adults, older adults) and temporal expectation (immediate effect condition, delayed effect condition). The results can be seen in Table 4 (upper part). The significant main effect of temporal expectation was modified by a significant interaction between age and temporal expectation. Correlation coefficients were higher in the immediate effect condition than in the delayed effect condition in younger adults (*p* <.001), but did not significantly differ in older adults (*p* =.09). Further, in the delayed effect condition correlation coefficients were lower in younger adults than in older adults (*p* =.016). No significant differences between younger and older adults were observed in the immediate effect condition (*p* =.24).

Further, we conducted regression analyses predicting the control ratings for the delays between 0 ms and 600 ms, separately for each participant. In Table 3 (right column) means of regression slopes depending on age (younger adults, older adults) and temporal expectation (immediate effect condition, delayed effect condition) are depicted. In both younger and older adults, a negative regression slope was observed in the immediate effect condition, whereas a positive regression slope was observed in the delayed effect condition. Again, regression slopes in the delayed effect condition were inverted by multiplying them with − 1 and an ANOVA with the between-participants factors age (younger adults, older adults) and temporal expectation (immediate effect condition, delayed effect condition) was performed. The results can be seen in Table 4 (lower part). The significant main effect of temporal expectation was modified by a significant interaction between age and temporal expectation. Regression slopes were higher in the immediate effect condition than in the delayed effect condition in younger adults (*p* <.001) but did not significantly differ in older adults (*p* =.86). Further, in the immediate effect condition regression slopes were higher in younger than in older adults (*p* =.005). No significant differences between younger and older adults were observed in the delayed effect condition (*p* =.19).

**Table 3:** Means of correlation coefficients (for the correlation between delay and control rating) and regression slopes (for the regression analyses predicting the control ratings for the delays between 0 ms and 600 ms) depending on age (younger adults, older adults) and temporal expectation (immediate effect condition, delayed effect condition)

age	temporal expectation	correlation coefficient	regression slope
younger adults	immediate effect condition	− 0.96	− 0.11
	delayed effect condition	0.59	0.05
older adults	immediate effect condition	− 0.93	− 0.07
	delayed effect condition	0.85	0.07

**Table 4:** Results of the ANOVAs with the between-participants factors age (younger adults, older adults) and temporal expectation (immediate effect condition, delayed effect condition) on correlation coefficients and regression slopes

	df_1_, df_2_	F	*p*	η^2^_*p*_
correlation coefficients				
age	1, 129	0.89	0.35	0.007
temporal expectation	1, 129	25.7	< 0.001	0.17
age x temporal expectation	1, 129	6.63	0.011	0.05
regression slopes				
age	1, 129	1.09	0.3	0.008
temporal expectation	1, 129	10.4	0.002	0.08
age x temporal expectation	1, 129	8.76	0.004	0.06

## Discussion

The aim of the present study was to investigate age-related differences in the use of temporal agency cues, i.e., temporal contiguity and temporal expectation, between younger (18–28 years) and older (65–75 years) adults. To this end, younger and older adults first completed adaptation blocks in which their action was consistently followed either by immediate effects (immediate effect condition) or by delayed effects (delayed effect condition). In subsequent test blocks, the delay between action and effect was systematically varied. Participants were told that during test blocks their action usually causes the effect, but that in some trials the effect is automatically generated by the computer independent of their action. Participants were asked to rate how much control they felt over the effect. In both, older and younger adults control ratings were higher for short action-effect delays compared to long action-effect delays in the immediate effect condition, whereas the opposite was the case in the delayed effect condition. Further, the correlation coefficients (between delay and control rating) and the regression slopes (from the regression analyses predicting control ratings for the delays between 0 ms and 600 ms) were negative for the immediate effect condition and positive for the delayed effect condition in younger and older adults. However, control ratings were lower in younger than in older adults at longer delays (300–600 ms) in the immediate effect condition, whereas no age differences appeared in the delayed effect condition. Further, correlation coefficients and regression slopes were higher in the immediate effect condition than in the delayed effect condition in younger adults but did not significantly differ in older adults. Also, in the immediate effect condition regression slopes were higher in younger than in older adults and in the delayed effect condition correlation coefficients were lower in younger than in older adults.

Higher control ratings for shorter than for longer action-effect delays in the immediate effect condition, and, vice versa, higher control ratings for longer than for shorter action-effect delays in the delayed effect condition in both older and younger adults were observed. This indicates that SoA is stronger the less the actual effect deviates from the expected effect. This is further corroborated by the result that correlation coefficients and regression slopes were negative in the immediate effect condition, but positive in the delayed effect condition in younger and older adults. Taken together, the data supports the hypothesis that temporal expectation is used as primary agency cue. If temporal contiguity would be used as primary agency cue one would expect higher control ratings for shorter than for longer delays in both the immediate and the delayed effect condition. As outlined in the introduction, the ideomotor framework offers a theoretical explanation for this finding. Through repeated action–effect experiences, associations between action and effect including their temporal characteristics are learned (Dignath et al., 2014; Dignath & Janczyk, 2017). Those temporal characteristics are retrieved upon action execution, strengthening SoA when the expected and actual delay match (Bart et al., 2024; Haering & Kiesel, 2015, 2016).

Most importantly, temporal expectation served as an agency cue in both age groups. Accordingly, these findings not only replicate previous results in younger adults (aged 19–39; Bart et al., 2024; Haering & Kiesel, 2015, 2016) but also extend them by showing that this cue is employed independently of age (at least up to the age of 75 as investigated in the present study). This is consistent with other studies, which showed that both younger and older adults rely on internal sensorimotor signals when inferring their SoA (Bart & Rieger, 2026; Mariano et al., 2024; Metcalfe et al., 2010). However, whereas earlier research primarily focused on age-related differences regarding the match between the predicted and actual identity of the effect as agency cue (Bart & Rieger, 2026; Metcalfe et al., 2010), the present study now emphasizes the temporal aspect (participants predicted the timing of the effect rather than its identity). Taken together, this suggests that internal sensorimotor cues regardless of the predicted feature (identity or temporal occurrence of the effect) may be used consistently across different stages of adulthood when inferring SoA.

Still, age-related differences between younger and older adults were also observed. In younger adults, correlations coefficients and regression slopes were higher in the immediate effect condition than in the delayed effect condition (see also Fig. 1 for the flatter curve in the delayed effect condition), whereas no such difference was observed in older adults. Thus, in younger adults, deviations from the expected timing predicted control ratings less well in the delayed effect condition than in the immediate effect condition (cf. Bart et al., 2024 for such results in younger adults). This may indicate that in younger adults SoA was not only affected by temporal expectation but also by temporal contiguity (as also reported by Bart et al., 2024), whereas older adults relied mainly on temporal expectation alone. In the immediate effect condition in younger adults, temporal expectation and temporal contiguity most likely reinforced each other: at short delays temporal contiguity was high and actual and predicted timing matched. This is also reflected in a steeper regression slope and in lower control ratings at long delays (300–600 ms) in younger than in older adults. Conversely, in the delayed effect condition in younger adults the same cues may have counteracted each other: at long delays the actual timing matched the predicted timing, but temporal contiguity was low. This is also reflected in a weaker correlation between control rating and delay in the delayed effect condition in younger compared to older adults. Taken together, older adults (at least up to the age of 75) rely solely on temporal expectation, as an internal sensorimotor agency cue, whereas younger adults use primarily temporal expectation but still integrate temporal contiguity, as an external sensory agency cue, when inferring SoA.

One may raise the question whether the temporal relation between action and effect was acquired in the same way across age groups during the adaptation blocks. For instance, age-related differences in memory (e.g. less fine-grained episodic encoding) may affect the formation of temporal action-effect associations, which could have contributed to the observed differences between age groups in the test blocks. However, the observed data pattern does not support this interpretation. In the delayed effect condition, regression slopes did not differ between younger and older adults, and in older adults regression slopes also did not differ between the immediate and delayed effect conditions. The main deviation from this pattern was observed in younger adults in the immediate effect condition, in which slopes were higher compared to both the delayed effect condition and to older adults in the immediate effect condition. Overall, this suggests that the observed differences are more likely driven by differences in the use of temporal contiguity as an agency cue in younger compared to older adults rather than by differences in the formation of temporal relations between action and effect.

The differences in cue integration, i.e. less reliance on external cues and more reliance on internal cues in older adults, are also consistent with previous results (Bart & Rieger, 2026; Cioffi et al., 2017; Mariano et al., 2024). As outlined in the introduction, this may result from age-related structural and functional changes in frontostriatal circuits that reduce the precision of external sensory signals, increasing reliance on less noisy internal sensorimotor cues (Wolpe et al., 2016; see also Mariano et al., 2024), such as an increased reliance on temporal prediction (Otsuka et al., 2025). However, external sensory cues are not always disregarded in older adults. For instance, Nobuksao et al. (2024) observed that older adults (65–83 yeas) may still use temporal contiguity as agency cue. As in this study temporal contiguity was the only available agency cue, one may argue that older adults use external sensory cues particularly when no other information (e.g. internal sensorimotor cues) is available.

It is also not necessarily the case that younger adults always integrate temporal contiguity. For instance, in the study of Haering and Kiesel (2015) it seems that younger adults only used temporal expectation as agency cue, but not temporal contiguity. It could be that in instances, in which temporal contiguity is quite high (e.g., delays varying from 0 ms to 250 ms as it was the case in the study of Haering & Kiesel, 2015), temporal expectation is the only agency cue used in younger adults, whereas in instances in which action and effect may be separated by longer delays (e.g., delays varying from 0 ms to 600 ms), younger adults attempt to integrate both temporal contiguity and temporal expectation (cf. Bart et al., 2024 for such a discussion).

One may raise however the question whether the results we take as evidence for temporal expectation as an agency cue really reflect internal predictive processes leading to an expectation or whether they rather reflect familiarity with a certain action-effect interval. Repeated exposure (and thus familiarity) may lead to processing fluency (Bornstein, and D’agostino, 1992). Thus, control ratings may reflect processing fluency rather than the comparison between expected and actual timing of an effect. However, recent accounts of processing fluency suggest that processing fluency itself is based on predictive processes (Servajean & Wiese, 2024), i.e., fluency arises when expectations and actual events match. At the core, this leads back to temporal expectations, even though the processes involved in bringing about the effect may be more complex than a simple match/mismatch comparison. More broadly, current accounts of SoA suggest that ratings are based on multiple sources of information, including temporal, predictive, and experiential information (Moore & Fletcher, 2012; Synofzik et al., 2008, 2009). From this perspective, the present findings may be viewed less as a matter of how to label specific agency cues, but rather as indicating that prior experience with action–effect relations contributes to control ratings across age groups.

On an even more general level one may raise the question how different agency cues ultimately contribute to one’s SoA, i.e. what underlying process transforms information from these cues into an explicit rating of control. This issue is rather unspecified in cue integration theories (Dewey & Knoblich, 2014; Lafleur et al., 2020). It is generally stated that the available cues are integrated into a common estimate of one’s SoA. From this perspective multiple agency cues may be weighted depending on their reliability and relevance in a given situation (Moore & Fletcher, 2012; Synofzik et al., 2008, 2009). However, this does not tell us much about the process of integration itself. For instance, rather than mere statistical integration, intermediate processes, such as judgement heuristics may play a role in weighing difference agency cues (Reddy, 2020). The present study cannot determine the exact pathway through which temporal cues influence SoA as it was not designed to do so. However, it highlights that temporal cues represent one source of information that is used to infer SoA.

Importantly, this does not imply that temporal cues were the only sources of information contributing to participants’ SoA ratings in the present study. SoA ratings remained above zero even when the temporal relationship between action and effect provided less support for SoA, suggesting that additional agency cues contributed to the overall level of SoA. For example, the key press in the present study provided somatosensory information that may have contributed to SoA ratings (see Cao et al., 2020 for the role of somatosensory response information in SoA), irrespective of the temporal relationship between action and effect. Thus, our findings suggest that age-related differences in SoA ratings reflect differences in how temporal agency cues are weighted, whereas other cues (e.g., response-related somatosensory information) may have contributed to the overall level of SoA across conditions and age groups.

One limitation of the present study is that participants experienced different violations of temporal expectancy across conditions. In the immediate effect condition, participants expected the effect to occur immediately, such that some trials in the test block involved effects occurring later than expected but no earlier-than-expected effects. In contrast, in the delayed effect condition, participants expected the effect after 600 ms, which represented the longest delay used, resulting in no later-than-expected but some earlier-than-expected effects. In the immediate effect condition, if an effect did not occur at the expected time point, participants may have increasingly expected it to occur as time elapsed and its conditional probability increased (i.e., a hazard effect; cf. Nobre et al., 2007). This hazard effect may serve as a third agency cue, which may interact with temporal contiguity and temporal expectations. This may have contributed to the observed differences between younger and older adults in the immediate effect condition (Droit-Volet et al., 2019). Additionally, as outline above, it has been speculated that the integration of temporal contiguity and temporal expectation as agency cues in younger adults may depend on the range of the different delays used (e.g. delays varying from 0 ms to 250 ms vs. delays varying from 0 ms to 600 ms, cf. Bart et al., 2024). Therefore, it would be interesting for future research to investigate at which delays individuals shift from relying solely on one temporal cue to integrating multiple temporal cues to infer SoA. Further, we only investigated healthy adults. However, certain diseases and disorders are associated with an increase (e.g. certain anxiety disorders like panic disorder, see Domschke et al., 2010 for a review) or decrease in interoceptive awareness (e.g. Parkinson’s disease, Ricciardi et al., 2016). Accordingly, future research may investigate whether and how depending on certain diseases or disorders the weighting of internal temporal cues relative to external temporal cues shifts when inferring SoA.

In conclusion, we showed that when temporal expectation and temporal contiguity are available agency cues, temporal expectation regarding the appearance of the effect is used as the primary agency cue in both younger and older adults. However, whereas in younger adults temporal contiguity between action and effect additionally impacts SoA, older adults do not appear to use this cue. Thus, taken together, both age groups rely on internal temporal sensorimotor agency cues (temporal expectation) when inferring SoA, but only younger adults additionally make use of external temporal sensory agency cues (temporal contiguity).

## Supplementary Information

Below is the link to the electronic supplementary material.

Supplementary Material 1 (DOC 100 KB)

## Data Availability

The data can be retrieved from the open science framework, https://osf.io/yghmb/.
