# Supplementary material for: Is timing everything? Age-related differences in the use of temporal agency cues
Source: Psychol Res. 2026 Oct 8;90(5):182. doi: 10.1007/s00426-026-02395-2 (PMC13649795; doi:10.1007/s00426-026-02395-2)
Supplement: Supplementary file 1 [file 426_2026_2395_MOESM1_ESM.doc]

SUPPLEMENTAL MATERIAL

**Is timing everything? Age-related differences in the use of temporal agency cues**

**Victoria K. E. Bart^1^ & Martina Rieger^1^**

1 Institute of Psychology, UMIT TIROL - Private University for Health Sciences and Health Technology, Hall in Tirol, Austria

Mail address of the corresponding author: victoria.bart@umit-tirol.at

1. **Comparisons of demographic data**

We compared whether the categorial variables (sex, handedness) differed depending on age (younger adults, older adults) and temporal expectation (immediate effect condition, delayed effect condition) using Pearson’s chi-squared tests. Results indicated that the distribution of *gender* did not differ significantly between the immediate effect condition and the delayed effect condition neither in younger adults, 𝛘^2^(1) = 0.02, *p* = .9, nor in older adults, 𝛘^2^(1) = 0.01, *p* = .92. Further, the distribution of gender did not differ significantly between younger and older adults, neither in the immediate effect condition, 𝛘^2^(1) = 0.06, *p* = .81, nor in the delayed effect condition, 𝛘^2^(1) = 0.04, *p* = .84. Similarly, results indicated that the distribution of *handedness* did not differ significantly between the immediate effect condition and the delayed effect condition neither in younger adults, 𝛘^2^(2) = 2.04, *p* = .36, nor in older adults, 𝛘^2^(2) = 2.05, *p* = .36. Further, the distribution of handedness did not differ significantly between younger and older adults, neither in the immediate effect condition, 𝛘^2^(2) = 2.06, *p* = .36, nor in the delayed effect condition, 𝛘^2^(2) = 1.96, *p* = .38.

Additionally, we compared whether the continuous variables (age in years, years of formal education, hours of sport per week, hours of cultural activity per week, hours of intellectual activity per week) differed depending on age (younger adults, older adults) and temporal expectation (immediate effect condition, delayed effect condition). To this end, ANOVAs with the between factors age (younger adults, older adults) and temporal expectation (immediate effect condition, delayed effect condition) were performed for each variable. The results of those ANOVAs can be seen in Table S1. As expected, for the variable *age in years* a significant main effect of age was observed, indicating that the age was higher in older adults than in younger adults. Further, there was a significant interaction between age and temporal expectation. Post-hoc tests indicated that in older adults, the age in years was (slightly) higher in the delayed effect condition (*M* = 69.8, *SD* = 3.64) than in the immediate effect condition (*M* = 67.6, *SD* = 2.68, *p* = .004), whereas no such difference was observed in younger adults (*p* = .77). However, this difference between conditions in older adults is unlikely to affect the overall conclusions, as all participants still fell within the same general age group. For the variable *hours of intellectual activity per week* (e.g. reading books, newspapers) there was a significant main effect of age, indicating that older adults spend more hours with intellectual activities (*M* = 10.85, *SD* = 0.9) than younger adults (*M* = 5.06, *SD* = 0.86). This may be explained by an increased free time due to retirement or by a desire to maintain cognitive health while aging. All other variables (*years of formal education, hours of sport per week,* and *hours of cultural activity per week*) did not differ significantly depending on age and temporal expectation.

**Table S1**
*Results of the ANOVAs with the between-participants factors age (younger adults, older adults) and temporal expectation (immediate effect condition, delayed effect condition) on age in years, years of formal education, hours of sport per week, hours of cultural activity per week, and hours of intellectual activity per week.*

|  | df_1_, df_2_ | *F* | *p* | η^2^_p_ |  |
| --- | --- | --- | --- | --- | --- |
| *Age in years* |  |  |  |  |  |
| age | 1, 129 | 7347 | <.001 | .98 |  |
| temporal expectation | 1, 129 | 3.82 | .053 | .029 |  |
| age x temporal expectation | 1, 129 | 5.54 | .02 | .041 |  |
| *Years of formal education* |  |  |  |  |  |
| age | 1, 129 | 1.55 | .22 | .012 |  |
| temporal expectation | 1, 129 | 0.55 | .46 | .004 |  |
| age x temporal expectation | 1, 129 | 0.34 | .56 | .003 |  |
| *Hours of sport per week* |  |  |  |  | |
| age | 1, 129 | 3.38 | .068 | .026 | |
| temporal expectation | 1, 129 | 1.55 | .22 | .012 | |
| age x temporal expectation | 1, 129 | 0.05 | .83 | <.001 | |
| *Hours of cultural activity per week* | | | | | |
| age | 1, 129 | 1.14 | .29 | .009 | |
| temporal expectation | 1, 129 | 1.74 | .19 | .013 | |
| age x temporal expectation | 1, 129 | 1.12 | .29 | .009 | |
| *Hours of intellectual activity per week* | | | | | |
| age | 1, 129 | 21.7 | <.001 | .14 | |
| temporal expectation | 1, 129 | 2.01 | .16 | .015 | |
| age x temporal expectation | 1, 129 | 0.12 | .74 | .001 | |

2. **Description of the tests used for the background neuropsychological screening**

For the background neuropsychological screening participants completed paper-pencil tests evaluating selective attention, verbal fluency, verbal and visual working memory, and spatial ability. To reduce the overall session length (so that the sessions would not become too exhausting, particularly for the older adults), some of those tests (as outlined in the corresponding section) were slightly abbreviated and simplified. Since the primary objective was to compare the cognitive performance between younger and older adults, rather than comparing the performance against norm values, using modified versions was not considered problematic.

*Selective attention:* A modified version of the revision of the d2 test of attention was employed (Brickenkamp et al., 2010). This is a cancellation test, in which participants are instructed to cross out target stimuli (the letter d with a total of two dashes placed above and/or below) embedded among similar distractor stimuli (the letter d with fewer or more than two dashes above and/or below them or the letter p with any number of dashes). In the original version, stimuli are arranged in 14 consecutive lines, with participants given 20 seconds per line before they are asked to move on to the next line. In the present study, only the first seven lines and a total time limit of two minutes (rather than separate time limits per line) were used. As outcome measures the number of processed target stimuli (independent of whether they were correctly crossed out or missed) as an index of processing speed, the concentration performance (calculated as the number of correctly crossed out target stimuli minus the number of incorrectly crossed out distractor stimuli), and error percent (reflecting the proportion of errors, i.e. incorrectly crossed out distractor stimuli and incorrectly omitted target stimuli, across all processed target stimuli) were calculated.

*Verbal Fluency:* Verbal fluency was assessed using the M-word subtest of the Regensburger word fluency test (Regensburger Wortflüssigkeitstest, Aschenbrenner et al., 2001). Participants were instructed to produce as many words as possible, beginning with the letter "M" within two minutes. Proper nouns, words sharing the same root, and words unlikely to appear in a German newspaper or book were not allowed. The outcome measure was the number of correctly generated words.

*Verbal working memory:* The digit span subtest of the Inventory for Memory Diagnostics (Inventar für Gedächtnisdiagnostik, Baller et al., 2006) was employed. Participants were required to repeat sequences of digits in the same order as presented orally by the experimenter. The task began with sequences of three digits, and sequence length increased by one digit after every two trials, up to a maximum of eight digits. If two sequences of the same length were repeated incorrectly, the test was discontinued. The outcome measure was the total number of correctly recalled digit sequences (the maximum value is 12).

*Visual working memory:* A modified version of the visual working memory subtest of the Inventory for Memory Diagnostics (Inventar für Gedächtnisdiagnostik, Baller et al., 2006) was employed. Participants were shown seven boxes arranged on a DIN A4 sheet, each containing a line varying in both position and orientation. They had 45 seconds to memorize position and orientation of the lines. Afterwards the same layout of now empty boxes was presented, and participants were instructed to reproduce the lines—matching both position and orientation—within 30 seconds. In contrast to the original version of the task, which involves three repetitions of the same task, the procedure was shortened, and participants only completed the task once. The outcome measure was the number of correctly reproduced lines (the maximum value is 7).

*Spatial ability:* Spatial ability was assessed with the spatial imagery subtest (subtest 9) of the Performance Testing System 50+ (Leistungsprüfsystem 50+, Sturm et al., 2015). Participants were presented with 40 different 3D-figures and had three minutes to indicate how many surfaces each figure had. As outcome measure the number of correctly solved items was used (the maximum value is 40).

Aschenbrenner, S., Tucha, O., & Lange, K. W. (2001). *Regensburger Wortflüssigkeits-Test.* Hogrefe.

Baller, G., Brand, M., Kalbe, E., & Kessler, J. (2006). *Inventar zur Gedächtnisdiagnostik.* Hogrefe.

Brickenkamp, R., Schmidt-Atzert, L., & Liepmann, D. (2010). *Test d2 – Revision.* Hogrefe.

Sturm, W., Willmes, K., & Horn, W. (2015). *LPS 50 +: Leistungsprüfsystem für 50-bis 90 Jährige.* Hogrefe.

3. **Comparisons of the data of the background neuropsychological screening**

We compared whether visual working memory, verbal working memory, verbal fluency, attention (number of processed target stimuli, concentration performance, error percent), and spatial ability differed depending on age (younger adults, older adults) and temporal expectation (immediate effect condition, delayed effect condition). To this end, ANOVAs with the between factors age (younger adults, older adults) and temporal expectation (immediate effect condition, delayed effect condition) were performed for each variable. The results of those ANOVAs can be seen in Table S2.

As can be expected, younger adults performed better than older adults on all neuropsychological tests (indicated by main effects of age), except *verbal fluency*. That is, they showed better *visual working memory*, better *verbal working memory*, better attention performance (higher *number of processed target stimuli,* higher *concentration performance,* and lower *error percent*), and better *spatial ability*. All other effects were not significant.

**Table S2**
*Results of the ANOVAs with the between-participants factors age (younger adults, older adults) and temporal expectation (immediate effect condition, delayed effect condition) on visual working memory, verbal working memory, verbal fluency, attention (number of processed target stimuli, concentration performance, error percent), and spatial ability.*

|  | df_1_, df_2_ | *F* | *p* | η^2^_p_ |  |
| --- | --- | --- | --- | --- | --- |
| *Visual working memory* |  |  |  |  |  |
| age | 1, 129 | 50.7 | <.001 | .28 |  |
| temporal expectation | 1, 129 | 0.004 | .95 | <.001 |  |
| age x temporal expectation | 1, 129 | 1.51 | .22 | .012 |  |
| *Verbal working memory* |  |  |  |  |  |
| age | 1, 129 | 20.3 | <.001 | .14 |  |
| temporal expectation | 1, 129 | 0.001 | .98 | <.001 |  |
| age x temporal expectation | 1, 129 | 0.14 | .71 | .001 |  |
| *Verbal fluency* |  |  |  |  | |
| age | 1, 129 | 2.86 | .093 | .022 | |
| temporal expectation | 1, 129 | 0.008 | .93 | <.001 | |
| age x temporal expectation | 1, 129 | 0.58 | .45 | .004 | |
| *Attention: number of processed target stimuli* | | | | | |
| age | 1, 129 | 137 | <.001 | .52 | |
| temporal expectation | 1, 129 | 0.31 | .58 | .002 | |
| age x temporal expectation | 1, 129 | 1.5 | .22 | .011 | |
| *Attention: concentration performance* | | | | | |
| age | 1, 129 | 163 | <.001 | .56 | |
| temporal expectation | 1, 129 | 0.001 | .98 | <.001 | |
| age x temporal expectation | 1, 129 | 1.41 | .24 | .011 | |
| *Attention: error percent* | | | | | |
| age | 1, 129 | 14.5 | <.001 | .1 | |
| temporal expectation | 1, 129 | 1.36 | .25 | .01 | |
| age x temporal expectation | 1, 129 | 0.002 | .97 | <.001 | |
| *Spatial ability* |  |  |  |  | |
| age | 1, 129 | 55.4 | <.001 | .3 | |
| temporal expectation | 1, 129 | 0.35 | .56 | .003 | |
| age x temporal expectation | 1, 129 | 0.14 | .71 | .001 | |
